# Th22 Cells/IL-22 Serves as a Protumor Regulator to Drive Poor Prognosis through the JAK-STAT3/MAPK/AKT Signaling Pathway in Non-Small-Cell Lung Cancer

**DOI:** 10.1155/2022/8071234

**Published:** 2022-05-28

**Authors:** Yinan Yao, Guangdie Yang, Guohua Lu, Jiani Ye, Luyun Cui, Zhu Zeng, Junjun Chen, Jianying Zhou

**Affiliations:** Department of Respiratory Medicine, The First Affiliated Hospital, College of Medicine, Zhejiang University, Hangzhou, China

## Abstract

The interaction of immune cells and cytokines in the tumor microenvironment affects the development and prognosis of tumors with an unclear potential regulatory mechanism. Recent studies have elucidated the protumor role of Th22 cells and its lineage-specific cytokine IL-22 in different human cancers. The present study is aimed at investigating the biological effect of Th22 cells/IL-22 and its molecular mechanism in the pathogenesis process of non-small-cell lung cancer (NSCLC). It was initially found that Th22 cells were enriched in the peripheral blood of NSCLC patients. The level of Th22 cells in peripheral blood mononuclear cells (PBMCs) was positively correlated with the TNM stage, lymph node metastasis, and clinical tumor biomarkers. Furthermore, IL-22 not only antagonized the apoptosis inducing and cell cycle arresting effect by chemotherapy and molecular targeted drugs on NSCLC cell lines but also promoted tumor cell proliferation and tumor tissue growth. Moreover, IL-22 activated the JAK-STAT3/MAPK/AKT signaling pathway, both *in vitro* and *in vivo*. Conclusively, the present results confirm that Th22 cells/IL-22 may serve as a negative immune regulator in lung cancer.

## 1. Introduction

Lung cancer is the most common malignant tumor worldwide and in China and has the highest incidence and cancer-related mortality [1–3]. Non-small-cell lung cancer (NSCLC) has a complex etiology and accounts for approximately 75-80% of lung cancers [4]. Although surgery, chemotherapy, radiotherapy, molecular targeting, and immunotherapy have made tremendous progress in NSCLC, the 5-year survival for advanced patients remains at less than 15%. Hence, there is a need to explore new treatment strategies for NSCLC.

In recent years, with the continuous increase in the use of tumor immunotherapies represented by PD-1/PD-L1, the tumor immune microenvironment, which regulates the invasion and metastasis of tumors, has received increasing attention. The interaction of immune cells and inflammatory cytokines between the tumor and its surrounding stroma affects the development and prognosis of tumors [5]. On one hand, exogenous inflammation can induce the imbalance of oncogenes and tumor suppressor genes, and in turn, induce the occurrence and progression of tumors. On the other hand, tumors can continuously generate a variety of inflammatory factors through autocrine and paracrine pathways. These factors attract inflammatory cells to participate in the immune regulation of host and tumor cells, including promoting proliferation, suppressing apoptosis, inducing angiogenesis, and obtaining immune tolerance [6–9]. Therefore, tumor-related immune inflammatory response is one of key points for tumor invasion and metastasis.

In order to identify the immune microenvironment involved in the potential regulation mechanism of NSCLC, focus was given on newly discovered T cell subsets, known as Th22 cells, and its lineage-specific cytokine, interleukin- (IL-) 22. Th22 cells were identified through the production of IL-22, without IFN-*γ*, IL-4, and IL-17, from other Th cells [10, 11]. Previous studies have revealed that Th22 cells and IL-22 are correlated with various tumors, including gastrointestinal cancer, hepatocellular carcinoma, and lung cancer [12–15]. Furthermore, it was reported that the level of IL-22 increases in tumor tissues and the malignant pleural effusion (MPE) of NSCLC hosts [16, 17]. Recently, Yu et al. detected the expression of IL-22 in new lesions and reported that this was significantly higher in the peripheral blood of recurrent NSCLC patients, when compared to that at baseline [18]. Hence, targeting Th22 cells and IL-22 may provide a new perspective for tumor therapy. However, although both pro- and antitumor effects have been reported, the relationship between Th22 cells/IL-22 and the prognosis of NSCLC patients remain unclear.

Therefore, the present study analyzed the expression of Th22 cells in NSCLC patients and its correlation with the tumor stage and metastasis. Furthermore, the biological role of IL-22, including affecting migration, cell cycle, antitumor drug resistance, and molecular mechanism, in NSCLC pathogenesis was investigated.

## 2. Materials and Methods

### 2.1. Patients and Blood Sample Preparation

The present study enrolled 31 patients (16 males and 15 females, age range: 40-78 years old) with pathologically proven primary lung cancer and 10 healthy controls (five males and five females, age range: 43-76 years old) from the First Affiliated Hospital of Zhejiang University Hospital between January 2018 and July 2019. Patients who previously received any therapy were excluded. Five milliliters of peripheral blood was collected from patients and healthy controls and anticoagulated with EDTA for the isolation of peripheral blood mononuclear cells (PBMCs). Then, this was analyzed by flow cytometry. The present study was approved by the ethics committee of the local hospital, and an informed consent was obtained from each patient or surrogate.

### 2.2. Cell Lines and Reagents

The human lung adenocarcinoma (AC) cell line A549 was bought from the National Collection of Authenticated Cell Cultures (Shanghai, China). The human lung AC cell line PC-9 was received as a gift from Professor Caicun Zhou (Shanghai Pulmonary Hospital, Shanghai, China). In the present study, all cells were cultured in RPMI-1640 (HyClone, Logan, UT, USA) supplemented with 10% fetal bovine serum (FBS; Gibco BRL Co., Ltd., Houston, TX, USA) at 37°C in 5% CO_2_. The human recombinant IL-22 was bought from PeproTech (Rocky Hill, NJ, USA). The IL-22 was dissolved in dimethyl sulfoxide (DMSO; St. Louis, MO, USA) at a concentration of 10 *μ*g/mL and stored at -20°C. The final concentration for the DMSO in the whole experiment was maintained at less than 0.1%. The p44/42 MAPK (ERK1/2), p-p44/42 MAPK (p-ERK1/2, T202/Y204), P38, p-P38 MAPK (Thr180/Tyr182), AKT, p-AKT (Thr308), JAK1, p-JAK1 (Tyr1034/1035), JAK2, p-JAK2 (Tyr1007/1008), STAT3 and p-STAT3 (Tyr705) antibodies, and GAPDH were purchased from Cell Signaling Technology (CST, Danvers, MA, USA).

### 2.3. Intracellular Flow Cytometry Analysis

The PBMCs were incubated for four hours with 50 ng/mL of phorbol 12-myristate 13-acetate (PMA; MultiSciences Biotech, Hangzhou, China), 1 *μ*g/mL of ionomycin (MultiSciences Biotech), and 500 ng/mL of monensin (eBioscience, San Diego, CA, USA) in a 6-well plate. The cells were initially stained for Pacific Blue-CD4 (eBioscience) on the surface. Then, these were fixed and permeabilized with IC Fixation/Permeabilization buffer (eBioscience), washed, and intracellularly stained with PE-Cy7-IL-17 (BioLegend, San Diego, CA, USA) and FITC-IL-22 (BioLegend). Finally, these cells were collected and analyzed using BD FACSVerse (BD Biosciences, NJ, USA). The flow cytometric data analysis was performed using the FlowJo software, version 10.0 (Tree Star Inc., San Carlos, CA, USA).

### 2.4. Wound Healing Assay

PC-9 and A549 cells were cultured in a 6-well plate until confluence. A wound was made by scraping the confluent cell monolayers using a 10 *μ*L pipette tip. Then, the cells were treated with different concentrations of IL-22 (0, 5, 10, 50, and 100 ng/mL) for 24 hours, and photographs of the wound gap were taken using a microscope (Olympus, Tokyo, Japan). The gap areas in each group were quantitatively evaluated by ImageJ (National Institutes of Health).

### 2.5. Migration Assay

PC-9 and A549 cells were starved overnight and added to the upper chamber (24-well Transwell chamber, pore size: 8 *μ*m; Corning, NY, USA) in serum-free media. Then, the lower chamber was filled with media that contain different levels of IL-22 (0, 5, 10, 50, and 100 ng/mL). After 48 hours of incubation at 37°C, the cells were fixed with methanol and stained with 0.1% crystal violet. Then, the number of cells in five random fields was counted.

### 2.6. Cell Apoptosis Assay

After treatment with gefitinib (5 *μ*mol/L, for PC-9) or cisplatin (10 *μ*mol/L, for A549) and IL-22 (0, 5, 10, 50, and 100 ng/mL) for 48 hours, the cells were harvested. Then, the FITC Annexin V Apoptosis Detection Kit (BD Pharmingen, NJ, USA) was used to detect the cell apoptosis, according to manufacturer's protocol. Briefly, cells were washed and resuspended in binding buffer at a concentration of 1 × 10^6^ cells/mL. Then, these cells were stained with 5 *μ*L of FITC Annexin V and 5 *μ*L of propidium iodide (PI) and kept in the dark for 15 minutes at room temperature. Finally, the cell apoptosis was performed using BD FACSVerse (BD Biosciences, San Jose, CA, USA).

### 2.7. Cell Cycle Assay

The serum-starved PC9 and A549 cells were treated with cisplatin (10 *μ*mol/L) and IL-22 (0, 10, 50, and 100 ng/mL) for 48 hours. Then, these cells were harvested, fixed in 70% ethanol, and stored at -20°C for 24 hours. Afterwards, these cells were stained with PI using the Cell Cycle and Apoptosis Analysis Kit (YEASEN, Shanghai, China) for 15 minutes at room temperature in the dark. The cell cycle was performed using BD FACSVerse (BD Biosciences, San Jose, CA, USA) and analyzed using the FlowJo software, version 10.1.

### 2.8. Western Blot Analysis

PC-9 and A549 cells were harvested and lysed with RIPA lysis buffer (Sangon Biotech, Shanghai, China). Then, the protein concentration was detected using the Pierce BCA Protein Assay Kit (Thermo Fisher Scientific, Rockford, IL, USA), according to manufacturer's instructions. Next, the protein was separated by sodium dodecyl-sulfate polyacrylamide gel electrophoresis (SDS-PAGE), transferred onto a polyvinylidene fluoride (PVDF) membrane (Millipore, Bedford, MA, USA), and incubated with the primary antibody overnight at 4°C. After incubating with the corresponding secondary antibody for one hour, the ECL Chemiluminescence Kit (FDbio, Hangzhou, China) was used to measure the density of the immunoreactive bands.

### 2.9. Xenograft Model Experiment

Female BALB/c-nude mice (3-4 weeks old, 16-18 g) were obtained from the Shanghai Experimental Animal Center (Chinese Academy of Sciences, Shanghai, China). Suspended A549 cells (5 × 10^5^ in 100 *μ*L of PBS per mouse) were subcutaneously injected into the right armpit of each mouse. When the tumor volume almost reached 50-100 mm^3^, these mice were randomly divided into two groups. Each group of mice was injected with IL-22 (4 *μ*g, daily; five days per week) or vehicle alone [19]. Then, the tumor size was measured every three days using a Vernier caliper, and the tumor volume was calculated using the following formula: *V* = (width^2^ × length)/2 [20]. At termination, these mice were sacrificed under anesthesia, and the entire tumor was harvested. All procedures were performed based on the Regulations for the Administration of Affairs Concerning Experimental Animals. These experiments were approved by the Experimental Animal Ethics Committee of Zhejiang University.

### 2.10. Statistical Analysis

All data were presented as the mean ± standard deviation (SD) of three independent experiments. The statistical significance was evaluated by Student's *t*-test or one-way ANOVA using the Prism 6.04 software (GraphPad Software Inc., San Diego, CA, USA). *P* < 0.05, *P* < 0.01, and *P* < 0.001 were considered statistically significant.

## 3. Results

### 3.1. Th22 Cells Are Enriched in Peripheral Blood of Patients with Lung Cancer

**Figure 1:**
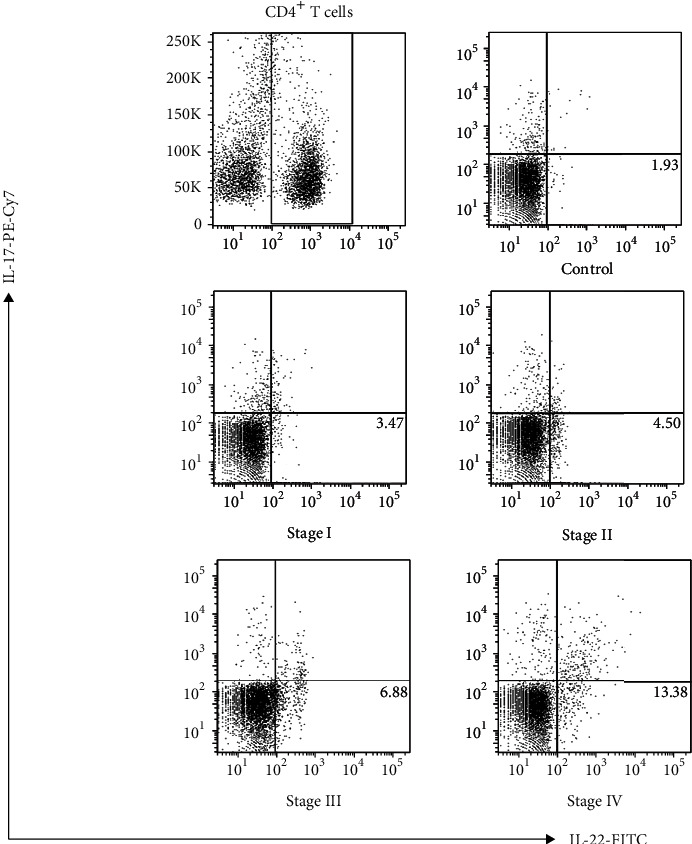
Th22 cells increased in PBMCs obtained from NSCLC patients. (a) The leukocytes isolated from the peripheral blood obtained from 31 patients with lung cancer and 10 healthy controls were analyzed by flow cytometry. The percentage of CD4 + IL-22 + IL-17- cells is shown. The numbers indicated in the lower right box represent the average values for the different groups.

In the present study, flow cytometry was performed to compare the level of Th22 cells in peripheral blood between patients with lung cancer and healthy controls. It was observed that patients with lung cancer had a higher percentage of CD4^+^IL-22^+^IL-17^−^ cells, when compared to healthy controls (controls vs. lung cancer patients: 1.93 ± 0.25% vs. 6.06 ± 0.65%, *P* < 0.0001; Figure 1).

### 3.2. The Level of Th22 Cells Is Correlated to the Pathological Stage and Clinical Tumor Biomarkers of Lung Cancer

**Figure 2:**
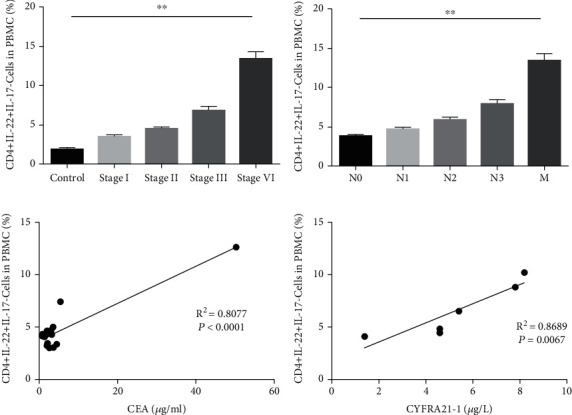
The level of Th22 cells in PBMCs is positively correlated with the pathological stage, lymph node metastasis, and clinical tumor biomarkers in lung cancer. (a) The relationship between Th22 cells in PBMCs and TNM stage (version 8) is shown. (b) The relationship between Th22 cells in PBMCs and metastasis is shown. The degree of lymph node metastasis was determined according to the 8th TNM classification. (c) The correlation between Th22 cells and serum CEA in adenocarcinoma (AC) patients. (d) The correlation between Th22 cells and serum CYFRA21-1 in squamous cell carcinoma (SCC) patients. The results were presented as mean ± standard deviation (SD). *n*: controls, 10; stage I, 10; stage II, 10; stage III, 6; stage IV, 5; ^∗∗^*P* < 0.01, compared to the previous group.

It was found that that the percentages of Th22 cells exhibited an upward trend in different stages of lung cancer patients, and this had a positive correlation with the TNM stage (Figure 2(a)).

The effect of Th22 cells on lymph node metastasis among the surgical specimens was also investigated, and it was observed that the levels of Th22 cells were positively correlated with the degree of lymph node metastasis and distal metastasis (Figure 2(b)).

In addition, the associations between the level of carcinoembryonic antigen (CEA) and frequency of Th22 cells in patients with AC and between the level of cytokeratin-19-fragment (CYFRA21-1) and frequency of Th22 cells in patients with squamous cell carcinoma (SCC) were, respectively, analyzed. The expression of CEA and CYFRA21-1 in serum obtained from patients with lung cancer was both positively associated with the frequency of Th22 cells (Figures 2(c) and 2(d)).

### 3.3. IL-22 Promotes Lung Cancer Cell Migration

**Figure 3:**
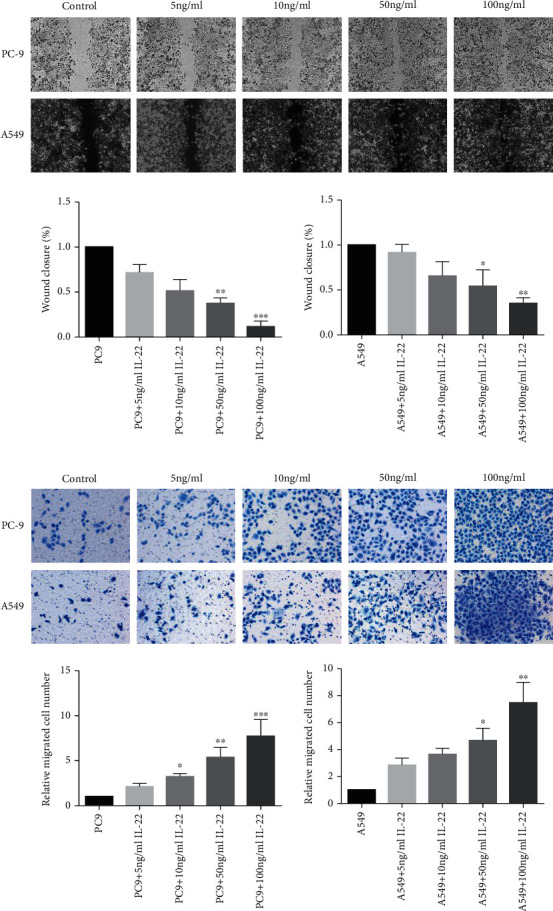
IL-22 promoted the cell migration of lung cancer cells. PC-9 and A549 cells were treated with IL-22 (0, 5, 10, 50, and 100 ng/mL) for 48 hours. The effect of IL-22 on tumor cell migration was evaluated by wound healing assay (scale bar = 100 *μ*m) (a) and Transwell assay (scale bar = 50 *μ*m) (b). The data are representatives of three independent experiments and presented as mean ± standard deviation (SD). ^∗^*P* < 0.05, ^∗∗^*P* < 0.01, and ^∗∗∗^*P* < 0.001, compared to the low concentration group.

Wound healing assay and the Transwell system were applied to determine the impact of IL-22 on cell migration. It was observed that the migration of both A549 and PC9 cells accelerated after IL-22 exposure, and the increase was dose-dependent after treatment with increasing concentrations (from 5 to 100 ng/mL, Figure 3(a)). Meanwhile, IL-22 obviously promoted the number of A549 and PC9 cells that transferred to the lower chamber (Figure 3(b)). Interestingly, the capacity of IL-22 in facilitating the migration on PC9 cells appeared to be more powerful, when compared to that for A549 (Figures 3(a) and 3(b)).

### 3.4. IL-22 Induces Antitumor Drug Resistance by Antagonizing Apoptosis

**Figure 4:**
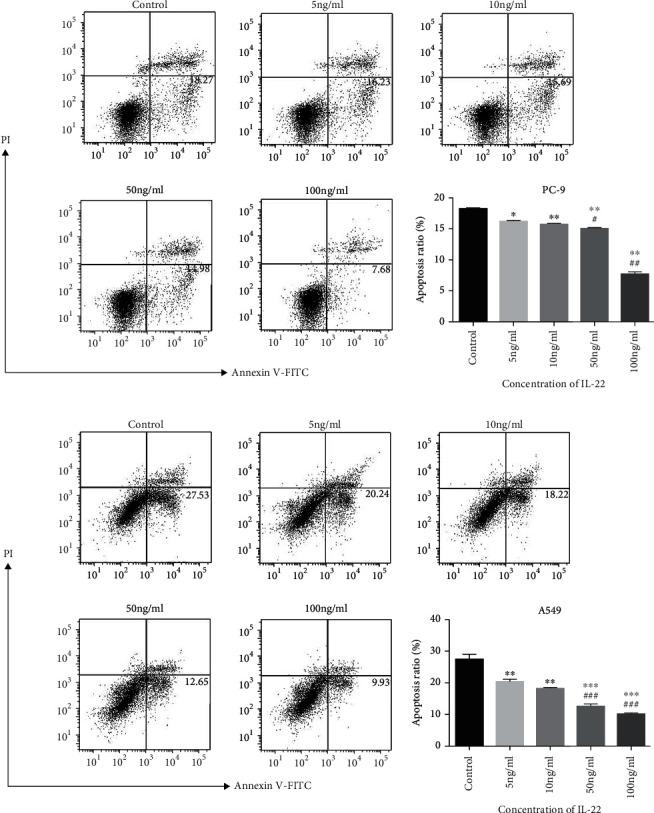
IL-22 antagonized the apoptosis induced by antitumor drugs on human lung NSCLC cells. PC-9 (a) was incubated with gefitinib (5 *μ*mol/L) and IL-22 (0, 5, 10, 50, and 100 ng/mL) for 48 hours, while A549 (b) was incubated with cisplatin (10 *μ*mol/L) and IL-22 (the same concentrations as PC-9) for 48 hours. Both cell lines were harvested, and the cell apoptosis was measured by flow cytometry. The data was presented as the mean ± standard deviation (SD) of triplicate samples. ^∗^*P* < 0.05, ^∗∗^*P* < 0.01, and ^∗∗∗^*P* < 0.001, when compared to the control group. ^#^*P* < 0.05, ^##^*P* < 0.01, and ^###^*P* < 0.001, when compared to the low concentration group.

In order to determine whether IL-22 induced antitumor drug resistance, PC9 cells were pretreated with gefitinib (5 *μ*mol/L), and A549 cells were pretreated with cisplatin (10 *μ*mol/L). Then, these were cultured with different concentrations of IL-22 for 48 hours. Cell apoptosis was detected by PI/FITC-Annexin V double staining. After 48 hours of IL-22 exposure, gefitinib-induced apoptotic PC9 cells and cisplatin-induced apoptotic A549 cells both significantly decreased, suggesting that IL-22 suppressed the antitumor sensitivity (Figures 4(a) and 4(b)). It is noteworthy that the reverse of IL-22 on the cell apoptosis treated by chemotherapy appeared to be more obvious, when compared to that by molecular targeted therapy (Figures 4(a) and 4(b)).

### 3.5. IL-22 Inhibits Cell Cycle Arrest Induced by Antitumor Drugs in Lung Cancer Cells

**Figure 5:**
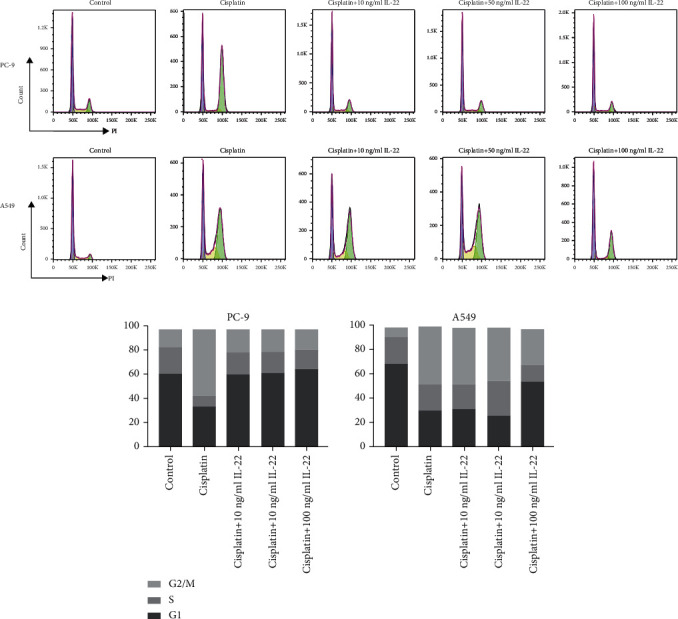
IL-22 inhibited the G2/M cell cycle arrest in NSCLC cells after cisplatin treatment. PC-9 (a) and A549 (b) were incubated with cisplatin (10 *μ*mol/L) and IL-22 (0, 5, 10, 50, and 100 ng/mL) for 48 hours. Both cell lines were collected, and the cell cycle analysis was performed by flow cytometry. The percentage of cells in each cell cycle stage was presented as the mean value of triplicate experiments.

Next, cell cycle assay was performed to determine whether IL-22 reversed the cell cycle arrest effect induced by antitumor drugs on the NSCLC cell lines. Cells were pretreated with gefitinib (5 *μ*mol/L) or cisplatin (10 *μ*mol/L) and exposed to serial doses of IL-22 for 48 hours. The flow cytometry analysis revealed that IL-22 markedly suppressed the G2/M arrest induced by cisplatin in both PC9 and A549 cells (Figures 5(a) and 5(b)), but not the gefitinib-mediated cell cycle arrest (data not shown). These results further confirm that IL-22 offsets the efficacy of antitumor drugs on NSCLC.

### 3.6. IL-22 Activates the JAK-STAT3/MAPK/AKT Signaling Pathway in Lung Cancer Cells

**Figure 6:**
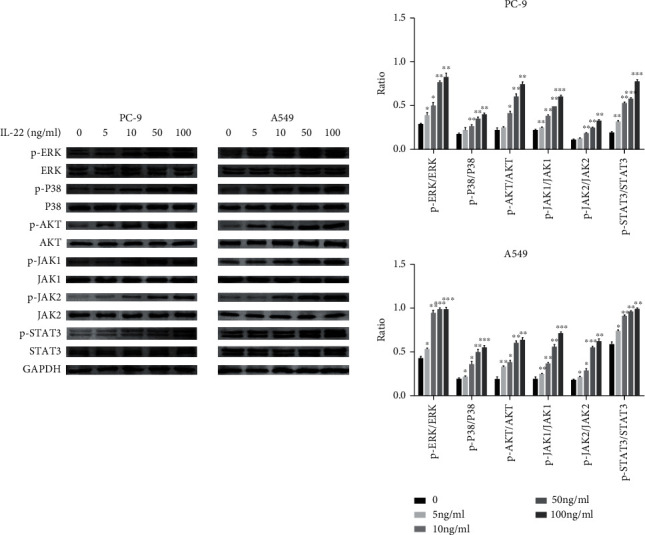
IL-22 activated the JAK-STAT3/MAPK/AKT signaling pathway in lung cancer cells. Cells were treated with IL-22 (0, 5, 10, 50, and 100 ng/mL) and harvested after 48 hours for western blot. The blots were representative of three experiments, and the results were quantified using Quantity One for comparisons among the control group and the different concentration groups. ^∗^*P* < 0.05, ^∗∗^*P* < 0.01, and ^∗∗∗^*P* < 0.001, when compared to the control group.

In order to determine the effect of IL-22 on a series of signaling pathways involved in cell cycle control, cell proliferation, and tumor growth, the activity of p38-MAPK/AKT/JAK-STAT was detected in PC9 and A549 cells. The western blot analysis revealed that IL-22 triggered the phosphorylation of p-ERK, p38, AKT, JAK1/2, and STAT3 in a dose-dependent manner (Figure 6).

### 3.7. IL-22 Exerts a Protumor Effect in A549 Xenograft Models

**Figure 7:**
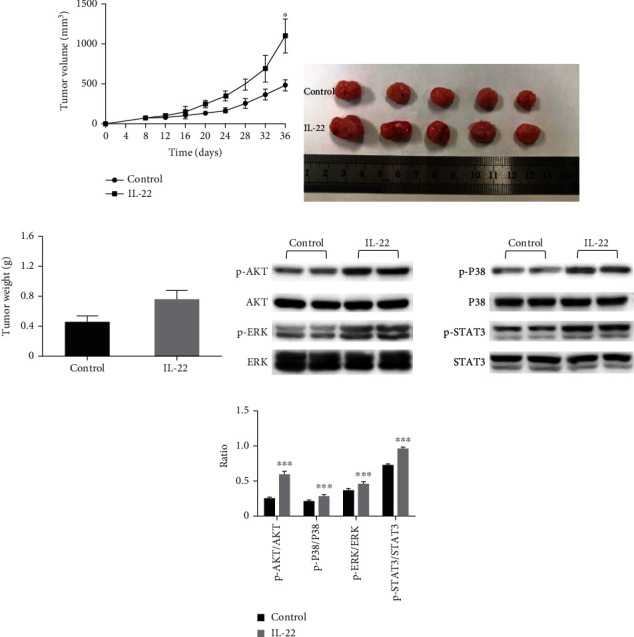
IL-22 accelerated the tumor growth in the xenograft models. A549 cells were subcutaneously injected into the right armpit of BALB/c nude mice. When the tumor volumes almost reached 50-100 mm^3^, these mice were randomly divided into two groups. Each group of mice was injected with IL-22 (4 *μ*g, daily; five days per week) or vehicle alone (*n* = 5). (a) The tumor volumes of mice were determined every three days after the onset of treatment. (b, c) Finally, these mice were sacrificed under anesthesia. Then, the entire tumor was resected, and the weight of the tumor was measured. (d, e) Subsequently, the harvested tumors were lysed, and western blot analysis was performed for p-AKT, AKT, p-p38, p38, p-ERK, ERK, p-STAT3, and STAT3. The data were presented as mean ± standard deviation (SD). The significant differences were compared with the controls and were indicated as ^∗^*P* < 0.05, ^∗∗^*P* < 0.01, and ^∗∗∗^*P* < 0.001.

In the present study, the A549 xenograft models were established to confirm the effect of IL-22 *in vivo*. It was found that there was a significant increase in tumor volume in IL-22-treated mice, when compared to control mice, at day 14 (Figure 7(a)), indicating that this can accelerate tumor growth in lung cancer-bearing mice. Furthermore, the IL-22 immediately shortened the survival of tumor-bearing mice (Figure 7(b)). Moreover, the phosphorylation of p38-MAPK/AKT/STAT3 was enhanced by IL-22 in the tumor samples, and these results were similar to those *in vitro* (Figure 7(c)).

## 4. Discussion

Immune cells and molecules in the tumor microenvironment are closely correlated to the prognosis of tumors in NSCLC. At the onset of tumors, positive immune regulatory cells, such as CD8^+^T cells, have predicted good prognosis in NSCLC patients [21], while negative immune regulatory cells, such as Tregs and tumor-associated macrophages (TAMs), may accelerate tumor progression [22, 23]. Therefore, there is an urgent need for more in-depth studies on the impact of tumor immune environment heterogeneity on tumor invasion and metastasis.

Th22 cells are a functional subset of CD4^+^ T cells and exhibit dramatic differences in the profile of altered genes, in contrast to Th1, Th2, and Th17 cells [10, 11]. IL-22 is the main effector of Th22 cells and is a new member of the IL-10 family [24]. Previous mainstream views have considered that Th22 cells and IL-22 have a promoting effect on tumors. However, to date, the correlation between Th22/IL-22, and clinical stage and prognosis remains controversial. Kobold et al. [14] reported that the level of IL-22 in tumor tissues is not correlated to tumor tissue size, lymph node invasion, distant metastasis, and survival prognosis. Another study revealed that the expression of IL-22 and its receptor in tumor tissues was higher, when compared to that in adjacent tissues, in lung cancer patients undergoing surgery [25]. In the present study, the expression of Th22 cells in PBMCs obtained from NSCLC patients and its correlation with tumor staging and metastasis were initially explored. It was found that the number of Th22 cells in PBMCs obtained from NSCLC patients significantly increased, when compared to those from healthy controls, and these had a positive correlation with the pathological TNM staging and lymph node metastasis. It was speculated that the tumor microenvironment may have induced the differentiation of Th22 cells in a tumor burden-dependent manner.

The role of IL-22 in promoting tumor invasion has been confirmed in a variety of cancers. A study revealed that the accumulation of intratumoral Th22 cells was most remarkable from stage II onwards in the patients with gastric cancer, but this was negatively correlated with survival [15]. Lately, Wang et al. have proven that IL-22 can significantly enhance the cell migration and invasion ability in triple-negative breast cancer (TNBC) cell lines *in vitro* [26]. Furthermore, according to previous studies, the speed of scratch-wound repair and the ability of tumor cells to pass through the membrane are facilitated after IL-22 exposure in NSCLC cell lines in a dose-dependent manner.

Tumor cells can evade apoptosis through disorders in the pathway of normal cell death. In order to determine the impact of IL-22 on NSCLC cell apoptosis, cisplatin and gefitinib were used as apoptosis inducers for A549 and PC-9 cells, respectively. As the dose of IL-22 increased, the apoptosis rate for both cell lines significantly decreased. Several other studies have revealed the same results. After transfecting the human IL-22 cDNA into lung cancer A549 and PG cells, the overexpression of IL-22 inhibited the chemotherapy-mediated apoptosis of A549 and PG cells through the activation of STAT3 and its downstream antiapoptotic proteins, such as Bcl-2 and Bcl-xl [16]. Li et al. reported that the expression of IL-22 significantly increased in pemetrexed-resistant A549 cells. After silencing the IL-22 gene by siRNA, the drug-resistant cell lines recovered the sensitivity to pemetrexed, and the apoptosis of A549 cells increased, accompanied by the activation of caspase-3 and the downregulation of Bcl-2 [27]. These above studies confirm that IL-22 can promote antitumor drug resistance and tumor cell apoptosis.

Previous studies have proven that IL-22 can activate multiple signal transduction pathways in cells, such as STAT3, MAPK, and AKT. Among these, IL-22 mainly stimulates the downstream target gene expression through the JAK/STAT pathway, such as cell cycle control and antiapoptotic genes [27, 28], and promotes tumor proliferation and metastasis. In the present study, it was found that the phosphorylation levels of STAT3, JAK1, JAK2, AKT, ERK, and P38 were significantly enhanced, both *in vitro* and *in vivo*, in a concentration-dependent manner. This confirms that IL-22 simultaneously activates the JAK-STAT3/MAPKs/AKT pathway in lung cancer.

The JAK/STAT3 pathway is the main signal transduction pathway of IL-22, and the activation of STAT3 promotes the invasion ability of malignant tumors. IL-22 augments the migration of rat mesenchymal stem cells through the IL-22RA1/STAT3 pathway [29]. Furthermore, IL-22 accelerates the proliferation and invasion of osteosarcoma cells by activating STAT3 [30]. Moreover, IL-22 promotes NSCLC cell development and STAT3 *via* IL-22R1 [18]. In addition, signaling pathways, such as MAPK and AKT, are also critical for IL-22 to participate in the regulation of tumor growth. In pancreatic cancer, the IL-22 secreted by innate immune cells activates the AKT signaling pathway to promote tumor metastasis [31]. Similarly, it was found that the IL-22 secreted by cancer-related fibroblasts elicits a protumor effect on NSCLC cell lines through the PI3K-Akt-mTOR signal pathway [25]. Furthermore, IL-22 upregulates the phosphorylation level of JNK in the pemetrexed-resistant A549 cell line, and this may be one of the mechanisms that promote the resistance of lung cancer cells to pemetrexed [32].

## 5. Conclusion

Th22 cells and its special cytokine IL-22 accelerate tumor progression and impair the prognosis of patients with NSCLC by antagonizing the apoptosis-inducing and cell cycle-arresting effect of antitumor drugs, promoting tumor cell migration and tumor tissue growth, and activating the JAK-STAT3/MAPK/AKT signaling pathway. These results confirm that Th22 cells/IL-22 may serve as a negative immune regulator in lung cancer and might provide a new therapeutic approach for NSCLC therapy.

## Data Availability

We state that if necessary, you can contact Yinan Yao or Jianying Zhou to obtain data (email: yaoyinan@zju.edu.cn; zjyhz@zju.edu.cn).
